# An Efficient Orthonormalization-Free Approach for Sparse Dictionary Learning and Dual Principal Component Pursuit

**DOI:** 10.3390/s20113041

**Published:** 2020-05-27

**Authors:** Xiaoyin Hu, Xin Liu

**Affiliations:** 1 Academy of Mathematics and Systems Science, Chinese Academy of Sciences, Beijing 100190, China; 2 School of Mathematical Sciences, University of Chinese Academy of Sciences, Beijing 100049, China; 3 State Key Laboratory of Scientific and Engineering Computing, Beijing 100190, China; liuxin@lsec.cc.ac.cn

**Keywords:** dual principal component pursuit, orthogonality constraint, sparse dictionary learning, stiefel manifold

## Abstract

Sparse dictionary learning (SDL) is a classic representation learning method and has been widely used in data analysis. Recently, the $\ell_{m}$-norm (m≥3,m∈N) maximization has been proposed to solve SDL, which reshapes the problem to an optimization problem with orthogonality constraints. In this paper, we first propose an $\ell_{m}$-norm maximization model for solving dual principal component pursuit (DPCP) based on the similarities between DPCP and SDL. Then, we propose a smooth unconstrained exact penalty model and show its equivalence with the $\ell_{m}$-norm maximization model. Based on our penalty model, we develop an efficient first-order algorithm for solving our penalty model (PenNMF) and show its global convergence. Extensive experiments illustrate the high efficiency of PenNMF when compared with the other state-of-the-art algorithms on solving the $\ell_{m}$-norm maximization with orthogonality constraints.

## 1. Introduction

In this paper, we focus on solving the optimization problem with orthogonality constraints:

(1) $$\begin{array}{cc} \min_{W\in R^{n\times p}}\; & f\left(W\right):=-\frac{1}{m}{∥W^{⊤}Y∥}_{m}^{m}\\ s.t.\; & W^{⊤}W=I_{p}, \end{array}$$

where *W* is the variable, $Y\in R^{n\times N}$ is a given data matrix, and $I_{p}$ denotes the identity matrix in $R^{p\times p}$. Besides, the $\ell_{m}$-norm is defined as ${∥Y∥}_{m}={\left[\sum_{i=1}^{n}\sum_{j=1}^{N}{\left(Y_{ij}\right)}^{m}\right]}^{\frac{1}{m}}$ with constant m∈(2,4]. For brevity, the orthogonality constraints $W^{⊤}W=I_{p}$ in (1) can be expressed as $W\in S_{n,p}:=\left\{W\in R^{n\times p}|W^{⊤}W=\;I_{p}\right\}$. Here, $S_{n,p}$ denotes the Stiefel manifold in real matrix space, and we call it the Stiefel manifold for simplicity in the rest of our paper.

The sparse dictionary learning (SDL) exploits the low-dimensional features within a set of unlabeled data, and therefore plays an important role in unsupervised representative learning. More specifically, given a data set $Y=\left[y_{1},y_{2},\cdots ,y_{N}\right]\in R^{n\times N}$ that contains *N* samples in $R^{n}$, SDL aims to compute a full-rank matrix $D\in R^{n\times p}$ named as dictionary, and an associated sparse representation $X=[x_{1},\cdots ,x_{N}]$ that satisfies

(2) Y=DX,

or equivalently, find a $W={D^{⊤}}^{-1}$ such that

(3) $$X=W^{⊤}Y.$$

As a result, the SDL can be solved by finding a $W\in R^{n\times n}$, which leads to a sparse $W^{⊤}Y$. Some existing works introduce the $\ell_{0}$-norm or $\ell_{1}$-norm penalty term to promote the underlying sparsity of $W^{⊤}Y$ and present various algorithms for solving the consequent optimization models, see the work in [1,2,3,4,5,6,7,8,9,10,11,12,13,14,15] for instance. Interested readers are referred to a recent paper [16] and the references therein. However, the $\ell_{1}$-norm minimization-based models are known to be sensitive with noise, and so far the existing approaches are not efficient enough for the purpose of solving real application problems which are often large-scale [17]. Consequently, a proper model with an efficient algorithm for SDL is desired, especially for the large-scale case.

Recently, an $\ell_{4}$-norm maximization model is proposed in [17], which can recover the entire dictionary in a single run. This new formulation is motivated by the fact that maximizing a higher-order norm promotes spikiness and sparsity at the same time. The authors of [17] demonstrate that the global minimizers of $\ell_{4}$-norm maximization with orthogonality constraints are very close to the true dictionary. Moreover, concaveness of the objective function in Equation (1) enables a fast fixed-point type algorithm, named matching, stretching, and projection (MSP). MSP achieves significant speedup compared with existing methods based on $\ell_{0}$-norm or $\ell_{1}$-norm penalty minimization. As maximizing any higher-order norm over a lower-order norm constraint leads to sparse and spiky solutions, Shen et al. [18] extend $\ell_{4}$-norm maximization technique to a generalized $l_{m}$-norm maximization (m≥3). In addition, the authors propose a gradient projection method (GPM) for solving it with guaranteed global convergence.

However, both MSP and GPM invoke polar decomposition to keep the feasibility in each iteration. As illustrated in  [19,20,21], orthonormalization lacks concurrency, which results in low scalability in column-wise parallel computing, particularly when the number of columns is large.

Several infeasible approaches have been developed to avoid orthonormalization. Gao et al. [19] propose the proximal linearized augmented Lagrangian method (PLAM) as well as its enhanced version, PCAL. Based on the merit function used in Gao et al. [19], Xiao et al. [21] propose an exact penalty model with a convex and compact auxiliary constraint, named PenC, for optimization problems with orthogonality constraints. The authors propose an approximated gradient method named PenCF for solving PenC and showed its global convergence and local convergence rate under mild conditions. The above-mentioned infeasible approaches do not require orthonormalization in each iteration. Numerical experiments illustrate the promising performance of these infeasible approaches with the existing state-of-the-art algorithms.

Although PCAL and PenCF avoid the orthonormalization process by taking infeasible steps, these approaches require additional constraints to restrict the sequence in a compact set in $R^{n\times p}$, which can undermine their overall efficiency. Therefore, to develop an efficient algorithm on solving SDL, an infeasible model without constraints is desired.

Similar to the $\ell_{1}$-norm penalty model for SDL, dual principal component pursuit (DPCP) aims to recover a tangent vector in $R^{n}$ from samples $Y=\left[y_{1},\cdots ,y_{n}\right]\in R^{n\times N}$ contaminated by outliers. Specifically, DPCP solves the following nonsmooth nonconvex optimization problem with a spherical constraint:

(4) $$\begin{array}{cc} \min_{W\in R^{n}}\; & {∥W^{⊤}Y∥}_{1}\\ s.t.\; & W^{⊤}W=1. \end{array}$$

Due to the ability on recovering an n−1 dimensional hyperplane from $R^{n}$, DPCP has wide applications in 3D computer vision, such as detecting planar structures in 3D point clouds in KITTI dataset [22,23] and estimating relative poses in multiple-view [24].

Existing approaches [25,26,27,28] for solving convex problem (4) are not scalable and not competent in high dimensional cases [29]. On the other hand, the Random Sampling and Consensus (RANSAC) algorithm [30] has been one of the most popular methods in computer vision for the high relative dimension setting. RANSAC alternates between fitting a subspace to a randomly sampled minimal number of points (n−1 in the case of DPCP) and measuring the quality of selected subspace by using the number of data-points close to the subspace. In particular, as described in [29], RANSAC can be extremely effective when the probability of sampling outlier-free samples inside the allocated time budget is large. Recently, Tsakiris and Vidal [31] introduce Denoised-DPCP (DPCP-d) by minimizing ${∥y-W^{⊤}Y∥}_{F}^{2}+\gamma {∥y∥}_{1}$ over the constraints $W^{⊤}W=1,y\in R^{N}$. In the same paper, Tsakiris and Vidal [31] propose an Iteratively-Reweighted-Least-Squares algorithm (DPCP-IRLS) for solving the non-convex DPCP problem (4). The authors illustrate that DPCP-IRLS can successfully handle 30% to 50% of outliers and showed its high efficiency compared with RANSAC. In addition, Zhu et al. [32] propose a projected subgradient-based algorithm named DPCP-PSGM, which exhibits great efficiency on reconstructing road-plane in the KITTA dataset. There are also some approaches using smoothing techniques to approximate the $\ell_{1}$-norm term such as Logcosh [8,33], Huber loss [34], pseudo-Huber [5], etc. Then, algorithms for minimizing a smooth objective function on a sphere can be applied. Nonetheless, these smoothing techniques often introduce approximation errors as the smooth objective functions usually lead to dense solutions. Qu et al. [35] and Sun et al. [8] propose a rounding step as postprocessing to achieve exact recovery [16] by solving a linear programming, which leads to addition computational cost.

The main difficulties in developing efficient algorithms are the nonsmoothness and nonconvexity in DPCP models. By observing the similarity between SDL and DPCP, we consider to adopt the $\ell_{m}$-norm maximization to reformulate DPCP as a smooth optimization problem on sphere.

### 1.1. Contribution

In this paper, we first point out that the DPCP problem can be formulated as the $\ell_{m}$-norm (m∈(2,4]) maximization (1) with p=1. Therefore, both SDL and DPCP can be unified as a smooth optimization problem on the Stiefel manifold.

Motivated by PenC [21], we propose a novel penalty function as the following expression,

(5) $$h\left(W\right):=f\left(W\right)-\frac{1}{2}\langle W^{⊤}W-I_{p},\Phi \left(W^{⊤}\nabla f\left(W\right)\right)\rangle +\frac{\beta }{6}{∥W∥}_{F}^{6}-\frac{\beta }{2}{∥W∥}_{F}^{2},$$

where β>0 is the penalty-parameter and Φ is the operator that symmetrizes the square matrix, defined by $\Phi \left(M\right)=\frac{M+M^{⊤}}{2}$. We show that h(W) is bounded from below, then the convex compact constraint in PenC can be avoided. Therefore, we propose the following smooth unconstrained penalty model for $\ell_{m}$-norm maximization (PenNM),

(6) $$\begin{array}{c} \min_{W\in R^{n\times p}}\;h\left(W\right). \end{array}$$

We prove that Equation (6) with m∈(2,4] is an exact penalty function of Equation (1) under some mild conditions. Moreover, when p=1, we verify that PenNM does not introduce any first-order stationary point other than those of Equation (1) and x=0. Based on the new exact penalty model, we propose an efficient orthonormalization-free first-order algorithm named PenNMF with no additional constraint. In PenNMF, we adopt an approximate gradient in each iterate instead of the exact one in which the second-order derivative of the original objective involves. The global convergence of PenNMF under mild conditions can be established.

The numerical experiments on synthetic and real imaginary data demonstrate that PenNMF outperforms PenCF and MSP/GPM in solving SDL, especially in large-scale cases. As an infeasible method, PenNMF shows superior performance when compared with MSP and GPM, which invoke an orthonormalization process to keep the feasibility. Moreover, when compared with PenCF, PenNMF also shows better performance, implying the benefits of avoiding the constraints in PenC. In our numerical experiments on DPCP, our proposed model (1) with p=1 shows comparable accuracy with $\ell_{1}$-norm based penalty model (4) on solving road-plane recovery in KITTA dataset. In some test examples, (1) can have even better accuracy than (4). Besides, PenNMF takes less CPU time while achieving comparable accuracy in reconstructing road-plane in KITTA dataset when compared with other state-of-the-art algorithms such as DPCP-PSGM and DPCP-d.

### 1.2. Notations and Terminologies

**Norms:** In this paper, ${∥\cdot ∥}_{m}$ denotes the element-wise *m*-th norm of a vector or matrix, i.e., ${∥A∥}_{m}={\left(\sum_{i=1}\sum_{j=1}{\left|A_{ij}\right|}^{m}\right)}^{1/m}$. Besides, ${∥\cdot ∥}_{F}$ denotes the Frobenius norm and $∥\cdot ∥$ denotes the 2-th operator norm, i.e., $∥A∥$ equals the maximum singular value of *A*. Besides, we denote $\sigma_{\min}\left(A\right)$ as the smallest singular value of a given matrix *A*. The operator A∘B stands for the Hadamard product of matrices *A* and *B* with the same size. |A| and $A^{\circ l}$ represent the component-wise absolute value and *l*-th power of matrix *A*, respectively. Besides, for two symmetric matrices *A* and *B*, A⪰B denotes that A−B is semi-positive definite, and A≻B denotes that A−B is positive definite.

**Optimality Condition:***W* is a first-order stationary point of (1) if and only if 

(7) $$\begin{array}{c} \left\{\begin{array}{ccc} \left(I_{n}-WW^{⊤}\right)\nabla f\left(W\right) & = & 0;\\ W^{⊤}\nabla f\left(W\right) & = & \nabla f{\left(W\right)}^{⊤}W;\\ W^{⊤}W & = & I_{p}. \end{array}\right. \end{array}$$

Besides, *W* is a first-order stationary point of PenNM if and only if ∇h(W)=0.

## 2. Model Description

In this section, we first discuss how to reformulate DPCP as an $\ell_{m}$-norm maximization with orthognoality constraints. To construct a orthonormalization-free algorithm, we minimize h(W) rather than directly solve (1). As an unconstrained penalty problem for (1), the model (6) may introduce additional infeasible first-order stationary points. Therefore, in this section, we characterize the equivalence between (1) and (6) to provide theoretical guarantees for our approach.

### 2.1. $\ell_{m}$-Norm Maximization for DPCP Problems

Based on the fact that maximization of a higher-order norm promotes spikiness and sparsity, we maximize the $\ell_{m}$-norm of ${\hat{W}}^{⊤}Y$ over the constraint ${\hat{W}}^{⊤}YY^{⊤}\hat{W}=1$. The model can be expressed as

$$\begin{array}{cc} \min_{\hat{W}\in R^{n}}\; & -\frac{1}{m}{∥{\hat{W}}^{⊤}Y∥}_{m}^{m}\\ s.t.\; & {\hat{W}}^{⊤}YY^{⊤}\hat{W}=1. \end{array}$$

Although with different constraints to (1), (4) can be reshaped to the formulation of (1). Let Y=RZ be the rank-revealing QR decomposition of *Y*, where $Z\in R^{n\times N}$ is an orthogonal matrix and $R\in R^{n\times n}$ is an upper-triangular matrix, and denote $W=R^{-T}\hat{W}$, then the optimization model can be reshaped as

(8) $$\begin{array}{cc} \min_{W\in R^{n}}\; & -\frac{1}{m}{∥W^{⊤}Z∥}_{m}^{m}\\ s.t.\; & W^{⊤}W=1. \end{array}$$

Clearly, problem (8) is a special case of (1) with p=1. Moreover, suppose $W^{*}$ is a global minimizer of (8), the solution for DPCP problem can be recovered by ${\hat{W}}^{*}=R^{-T}W^{*}$. The detailed framework for solving DPCP by $\ell_{m}$-norm maximization is presented in Algorithm 1.
**Algorithm 1** Framework for Solving DPCP by $\ell_{m}$-Norm Maximization.**Require:** Data matrix $Y\in R^{n\times N}$ 1: Perform QR-factorization for *Y*. Namely, Y=RZ where *R* is upper-triangular matrix and $Z\in R^{n\times N}$ is orthogonal matrix; 2: Compute the solution $\hat{W}$ for (1); 3: Return $W^{*}=R^{-T}\hat{W}$


### 2.2. Equivalence

In this subsection, we first derive the expression for ∇f(W) and ∇h(W).

**Proposition** **1.** *The gradient and the Hessian of f(W) can be expressed as*

$$\begin{array}{c} \nabla f\left(W\right)=Y\left[{|Y^{⊤}W|}^{\circ (m-1)}\circ \mathrm{sign}\left(\left(Y^{⊤}W\right)\right)\right];\\ \nabla^{2}f\left(W\right)\left[D\right]=\left(m-1\right)Y\left[{\left(Y^{⊤}W\right)}^{\circ (m-2)}\circ \left(Y^{⊤}D\right)\right], \end{array}$$

*respectively. Moreover, the gradient of h(W) can be formulated as*

$$\begin{array}{cc} \nabla h(W)= & \nabla f\left(W\right)\left(\frac{3}{2}I_{p}-\frac{1}{2}W^{⊤}W\right)-W\Phi \left(W^{⊤}\nabla f\left(W\right)\right)-\frac{1}{2}\nabla^{2}f\left(W\right)\left[W\left(W^{⊤}W-I_{p}\right)\right]\\ \; & +2\beta WW^{⊤}W\left(W^{⊤}W-I_{p}\right)+\beta W\left(W^{⊤}WW^{⊤}W-I_{p}\right). \end{array}$$

**Proof.**  From the work in [17] we have $\nabla f\left(W\right)=Y\left[{|Y^{⊤}W|}^{\circ (m-1)}\circ sign\left(Y^{⊤}W\right)\right]$. Based on the expression for ∇f(W), the Hessian of *f* can be expressed as $\nabla^{2}f\left(W\right)\left[D\right]=\left(m-1\right)Y\left[\left(Y^{⊤}D\right)\circ {\left(Y^{⊤}W\right)}^{\circ (m-2)}\right]$. As a result, $\nabla^{2}f\left(W\right)\left[W\left(W^{⊤}W-I_{p}\right)\right]=\left(m-1\right)Y{\left(Y^{⊤}W\right)}^{\circ (m-1)}Y\left(W^{⊤}W-I_{p}\right)$.

Therefore, based on ([21], Equation 2.8), the gradient of h(W) can be formulated as

$$\begin{array}{cc} \nabla h(W)= & \nabla f\left(W\right)\left(\frac{3}{2}I_{p}-\frac{1}{2}W^{⊤}W\right)-W\Phi \left(W^{⊤}\nabla f\left(W\right)\right)-\frac{1}{2}\nabla^{2}f\left(W\right)\left[W\left(W^{⊤}W-I_{p}\right)\right]\\ \; & +2\beta WW^{⊤}W\left(W^{⊤}W-I_{p}\right)+\beta W\left(W^{⊤}WW^{⊤}W-I_{p}\right) \end{array}$$

 □

With the expression for ∇h(W), we can establish the equivalence between (1) and our proposed model, (6). The equivalence is illustrated in Theorem 4, and the main body of the proofs is presented in Appendix A.

**Theorem** **2.** **(First-order equivalence)** *Suppose $\beta \geq \left(4m+8\right){∥Y∥}_{F}^{m}$ and $\tilde{W}$ is a first-order stationary point of (6), then either ${\tilde{W}}^{⊤}\tilde{W}=I_{p}$ holds, which further implies that $\tilde{W}$ is a first-order stationary point of problem (1), or the inequality $\sigma_{\min}\left({\tilde{W}}^{⊤}\tilde{W}\right)\leq \frac{\left(2m+4\right){∥Y∥}_{F}^{m}}{\beta }$ holds.*

Theorem 2 characterizes the relationship between the first-order stationary points of (1) and those of (6). Namely, the penalty model only yields the first-order stationary points other than those of the original model (1) far away from the Stiefel manifold. When p=1, we can derive a stronger result on those additional first-order stationary points produced by the penalty model in Corollary 3.

**Corollary 3.**  **(Stronger first-order equivalence for p=1)** *Suppose p=1 in (1), $\beta \geq \left(4m+8\right){∥Y∥}_{F}^{m}$, and $\tilde{W}$ is a first-order stationary point of (6), then either ${\tilde{W}}^{⊤}\tilde{W}=I_{p}$ holds, which further implies that $\tilde{W}$ is a first-order stationary point of problem (1), or $\tilde{W}=0$.*

Theorem 2 characterizes the equivalence between (1) and (6) in the sense that all the infeasible first-order stationary points of (6) is relatively far away from the constraint $W^{⊤}W=I_{p}$. Besides, Corollary 3 shows that when p=1, the only infeasible first-order stationary point of (6) is 0. Therefore, when we achieve a solution near the constraint $W^{⊤}W=I_{p}$ by solving (1), we can conclude that *W* is a first-order stationary point of (1). Instead of directly solving (1), we can compute the first-order stationary point of (6) and thus avoid intensive orthonormalization in the computation.

## 3. Algorithm

### 3.1. Global Convergence

In this section, we focus on developing an infeasible approach for solving (6). The calculation of the gradient of h(W) is involved with the second-order derivative, which is typically even more expensive than the iterations in MSP/GPM. Therefore, we consider to solve (6) by an approximated gradient descent algorithm. Let $D\left(W\right):=\nabla f\left(W\right)-W\Phi \left(W^{⊤}\nabla f\left(W\right)\right)+\beta W\left(W^{⊤}WW^{⊤}W-I_{p}\right)$ be the approximation for the gradient of h(W), we present the detailed algorithm as Algorithm 2.
**Algorithm 2** First-Order Method for Solving (6). (PenNMF)**Require:**$f:R^{n\times p}\mapsto R$, β>0; 1: Randomly choose $W^{0}$ satisfies ${W^{0}}^{⊤}W^{0}=I_{p}$, set k=0; 2: **while** not terminate **do** 3:  Compute inexact gradient
$$D\left(W^{k}\right)=\nabla f\left(W^{k}\right)-W^{k}\Phi \left({W^{k}}^{⊤}\nabla f\left(W^{k}\right)\right)+\beta W^{k}\left({W^{k}}^{⊤}W^{k}{W^{k}}^{⊤}W^{k}-I_{p}\right);$$ 4:  Compute stepsize $\eta^{k}$; 5:  $W^{k+1}=W^{k}-\eta^{k}D\left(W^{k}\right)$; 6:  k++; 7: **end while** 8: Return $W^{k}$

Next, we establish the convergence of PenNMFin Theorem 4, which illustrates the global convergence and worst-case convergence rate of PenNMF under mild conditions. The main body of the proof is presented in Appendix B.

**Theorem** **4.** **(Global convergence)** *Suppose $\delta \in \left(0,\frac{1}{3}\right]$ and $\beta \geq \max\left\{228m{∥Y∥}_{F}^{m},\frac{32}{\delta }{∥Y∥}_{F}^{m}\right\}$. Let $\left\{W^{k}\right\}$ be the iterate sequence generated by PenNMF, starting from any initial point $W^{0}$ satisfying $\left|\right|{W^{0}}^{⊤}W^{0}-I_{p}{\left|\right|}_{F}^{2}\leq \frac{1}{8}\delta$, and the stepsize $\eta_{k}\in \left[\frac{1}{2}\bar{\eta },\bar{\eta }\right]$, where $\bar{\eta }\leq \frac{1}{2M_{1}}$. Then, $W^{k}$ weakly converges to a first-order stationary point of (1). Moreover, for any k=1,2,⋯, the convergence rate of PenNMF can be estimated by*

(9) $$\min_{0\leq i\leq k}{∥D(W^{i})∥}_{F}\leq \sqrt{\frac{8m{∥Y∥}_{F}^{m}+2\beta \delta }{\bar{\eta }\left(k+1\right)}}.$$

### 3.2. Some Practical Settings

As illustrated in Algorithm 2, the hyperparameters in PenNMF are the penalty parameter β and stepsize $\eta^{k}$. In the theoretical analysis for PenNMF, the upper bound of $\eta^{k}$ adopted in Theorem 4 is too restrictive in practice. There are many adaptive stepsize for first-order algorithms, and here we consider the Barzilai–Borwein (BB) stepsize [36],

(10) $$\eta_{BB1,k}=\frac{\langle S_{k},Y_{k}\rangle }{\langle Y_{k},Y_{k}\rangle },\;\eta_{BB2,k}=\frac{\langle S_{k},S_{k}\rangle }{\langle S_{k},Y_{k}\rangle },$$

and alternating Barzilai–Borwein (ABB) stepsize [37],

(11) $$\eta_{ABB}^{k}=\left\{\begin{array}{cc} \eta_{BB1}^{k} & mod(k,2)=1\\ \eta_{BB2}^{k} & mod(k,2)=0, \end{array}\right.$$

where and $S_{k}=W^{k}-W^{k-1}$, $Y_{k}=\nabla h\left(W^{k}\right)-\nabla h\left(W^{k-1}\right)$. We suggest to choose the stepsize $\eta^{k}$ as ABB stepsize in PenNMF, and we test PenNMF with ABB stepsize in our numerical experiments.

Another parameter is β, which controls the smooth penalty term in h(W). Similarly, the lower-bound for β in Theorem 4 is too large to be practical. In our numerical examples, we uses the constant $s:={∥\nabla f(W^{0})∥}_{F}$, which is an approximation to ${∥\nabla^{2}f\left(W^{0}\right)∥}_{F}$, as an upper-bound for β. According to the work in [21], we suggest to choose the penalty parameter by $\beta =0.01{∥\nabla^{2}f\left(W^{0}\right)∥}_{F}$.

Additionally, to achieve high accuracy in feasibility, we perform the polar factorization to the final solution generated by PenCF and PenNMF as the default postprocess. More precisely, when we compute the final solution $W^{k}$ by PenNMF, we can compute its rank-revealing singular-value decomposition $W^{k}=U^{k}\Sigma^{k}{V^{k}}^{⊤}$ and return ${\hat{W}}^{k}:=U^{k}{V^{k}}^{⊤}$. Using the same proof techniques in [21], our postprocess leads to decrease in feasibility as well as the functional value. Moreover, the numerical experiments in [19] show that the introduced orthonormalization process results in little changes in ∇h(W). Therefore, we suggest to perform the described postprocess for PenNMF.

## 4. Numerical Examples

In this section, we present our preliminary numerical examples. We compare our algorithm with some state-of-the-art algorithms on SDL and DPCP problems, which are formulated as (1) and (8), respectively. Then, we observe the performance of our algorithm under different selections of parameters, and then choose the default setting. All the numerical experiments in this section are tested on an Intel(R) Core(R) Silver 4110 CPU @ 2.1 GHz, with 32 cores and 394 GB of memory running under Ubuntu 18.04 and MATLAB R2018a.

### 4.1. Numerical Results on Sparse Dictionary Earning

In this subsection, we mainly compare the numerical performance of PenNMF with some state-of-the-art algorithms on SDL. As illustrated in Table 2 in [17], MSP is significantly faster than the Riemannian subgradient [3] and Riemannian trust-region method [8]. Therefore, to have a better illustration on the performance of PenNMF, we compare PenNMF with state-of-the-art algorithms on solving (1), which is a smooth optimization problem with orthogonality constraints. We first select two state-of-the-art algorithms on solving optimization problems with orthogonality constraints. One is Manopt [38,39], a projection-based feasible method. In our numerical test, we choose nonlinear conjugate gradient with inexact linear-search strategy to accelerate Manopt. Another one is PenCF [21], which is an infeasible approach for optimization problems with orthogonality constraints. In our algorithms we choose to apply Alternating Bzarzilar–Borwein stepsize to accelerate PenNMF, and uses all parameters as default setting described in [21]. Besides, we test the MSP algorithm [17] and GPM algorithm [18]. It is worth to mention that when m=4, the MSP and GPM are actually the same. According to the numerical examples in [18], m=3 has better recovery quality than the case m=4. Therefore, in our numerical experiments, we test the mentioned algorithms on the case where m=3.

The stopping criteria for Manopt, MSP/GPM is ${∥\nabla f\left(W^{k}\right)-W^{k}\Lambda \left(W^{k}\right)∥}_{F}\leq 10^{-2}$, while the stopping criteria for PenCF and PenNMF is ${∥\nabla h(W^{k})∥}_{F}\leq 10^{-2}$. Besides, the max iteration for all compared algorithms is set as 200.

In all test examples, we randomly generate the sparse representation *X* by $X^{*}=\mathrm{randn}\left(n,N\right).*\left(\mathrm{randn}\left(n,N\right)<0.3\right)$ and the dictionary $W^{*}$ by randomly selecting a point on Stiefel manifold. Then, the original data matrix *Y* is constructed by $Y_{o}={W^{*}}^{⊤}X^{*}$. To test the performance of all compared algorithms, we add different types of noise to $Y_{o}$. We first fix the level of noise θ=0.3 and choose *n* from 20 to 100. Then, we test the performance of compared algorithms with different types of noisy while fix n=50. In our numerical tests, the “Noise” denotes the Gaussian Noise, where *Y* is constructed by $Y=Y_{o}+\theta \cdot \mathrm{randn}\left(n,N\right)$. Besides, the term “Outliers” denotes the Gaussian outliers, where $\mathrm{outliers}=\mathrm{randn}\left(n,\mathrm{round}\left(\theta m\right)\right);Y=\mathrm{cat}\left(2,Y_{o},\mathrm{outliers}\right);$. Additionally, the term “Corruption” refers to the Gaussian corruption to $Y_{o}$, which is achieved by $\mathrm{rademacher}=\left(\mathrm{rand}\left(n,m\right)<0.5\right)*2-1;Y=Y_{o}+\left(\mathrm{rand}\left(n,m\right)<\theta \right).*\mathrm{rademacher}$. Besides, the term ’CPU time’ denotes the averaged run-time, while the term ’Error’ denotes the $1-{∥{\hat{W}}^{⊤}W^{*}∥}_{4}^{4}$, where $\hat{W}$ denotes the final output of all the compared algorithm.

**Figure 1:**
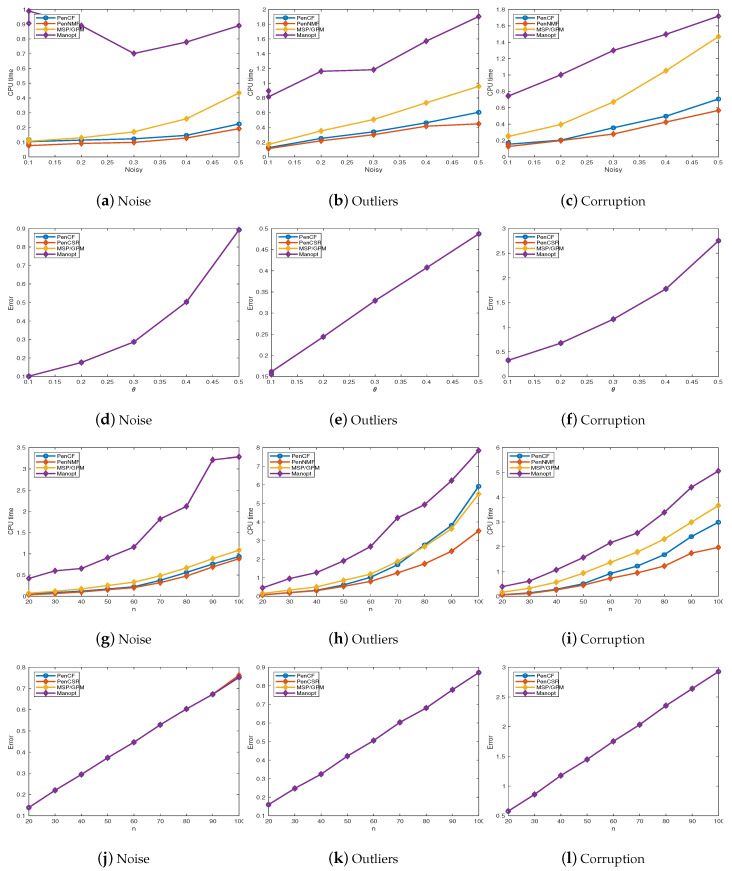
A detailed comparison among MSP, Manopt, PenCF, and PenNMF. (**a**)–(**c**) a comparison with different level of noisy on CPU time; (**d**)–(**f**) a comparison with different level of noisy on errors; (**g**)–(**i**) a comparison with different *n* on their CPU time; (**j**)–(**l**) a comparison with different *n* on their errors. The errors are evaluated by $1-{∥{\hat{W}}^{⊤}W^{*}∥}_{4}^{4}$, where $\hat{W}$ denotes the final output of all the compared algorithm.

The numerical results are listed in Figure 1. From Figure 1d–f, j–l we conclude that all these compared algorithms achieve almost the same accuracy in all the cases. Besides, for Gaussian noise, the performance of PenNMF is comparable to MSP/GPM algorithm and outperforms Manopt. Moreover, with Gasuuain outliers and Gaussian corruption, the performance of PenNMF is better than PenCF, MSP/GPM, and Manopt. One possible explanation is that for Manopt invokes computing the Riemannian gradient, line-search in each iteration, resulting in higher computational complexity than MSP/GPM. Besides, the infeasible approaches overcome the bottleneck in the orthonormalization process in Manopt and MSP/GPM, and thus achieve comparable performance to MSP/GPM. Additionally, PenCF solves a constrained model by taking approximated gradient descent steps, while in PenNMF the model is an unconstrained one. The absence of constraint helps to improve the performance of PenNMF.

**Figure 2:**
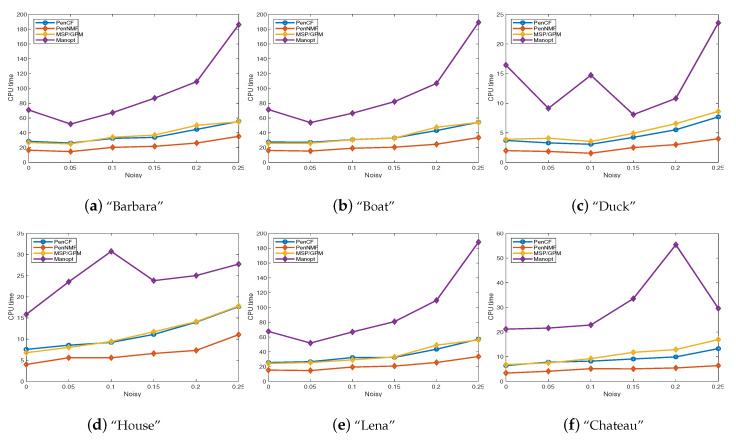
The CPU time of PenCF, PenNMF, MSP/GPM, and Manopt on computing the dictionary. (**a**) Barbara, $Y\in R^{256\times 247,009}$; (**b**) Boat, $Y\in R^{256\times 247,009}$; (**c**) Duck, $Y\in R^{192\times 62,001}$; (**d**) House, $Y\in R^{256\times 58,081}$; (**e**) Lena, $Y\in R^{256\times 247,009}$; (**f**) Chateau, $Y\in R^{192\times 146,633}$.

**Figure A1:**
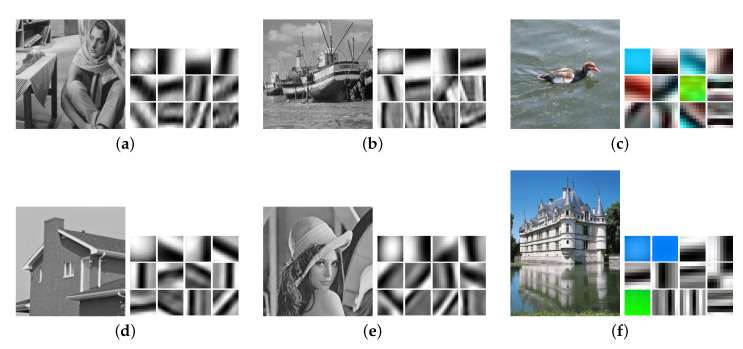
The top 12 bases computed from all patches of the test images without noise by PenNMF. (**a**) “Barbara“; (**b**) “Boat”; (**c**) “Duck“; (**d**) “House”; (**e**) “Lena“; (**f**) “Chateau”.

Besides testing on synthetic datasets, we also perform extensive experiments to verify the performance of PenNMF on real imagery data. A classic application of dictionary learning involves learning sparse representations of image patches [40]. In this paper, we extend the experiments in [17] to learn patches from grayscale and color images. Based on the 512×512 grayscale image “Barbara”, we construct the clean data matrix $Y_{o}$ by vectorizing each 16×16 patches from it. Then, we use the same approach to construct the clean data matrix *Y* from 512×512 grayscale images “Boat” and “Lena”, together with a 256×256 grayscale image ”House”. In “Barbara”, “Boat”, and “Lena”, the clean data matrix $Y\in R^{256\times 247,009}$, and the data matrix from “House” satisfies $Y\in R^{256\times 58,081}$. Besides, we construct the matrix $Y\in R^{192\times 62,001}$ by vectorizing the 8×8×3 patches from the 256×256 RGB image “Duck”. In such setting, all the compared algorithms recover the dictionary for all three channels simultaneously rather than learn them once for each channel in “Duck”. Such approach is aslo applied to generate the data matrix in $R^{192\times 146,633}$ from 338×450 RGB image “Chateau”. We run MSP/GPM, PenNMF, PenCF, and Manopt with m=3 to compute the dictionary from $Y=Y_{o}+\theta \cdot \mathrm{randn}\left(n,N\right)$ with different level of noise, where $Y_{o}$ is generated in the same manner as our first numerical experiment and has the same size as these patched figures. The numerical results are presented in Figure 2 and Figure A1. In all experiments, PenNMF takes less time than PenCF, MSP/GPM, and Manopt, which further illustrate the high efficiency of PenNMF in tackling the real imagery data, especially in the large-scale case.

### 4.2. Dual Principal Component Pursuit

**Figure 3:**
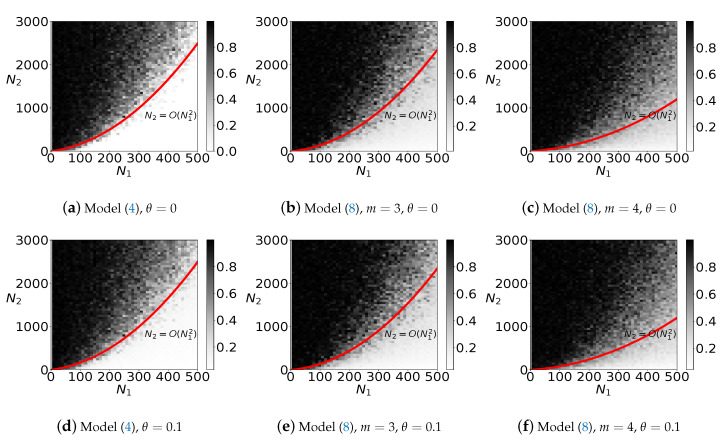
A comparison between the models (8) and (4) on the average recovery error $\sqrt{1-{\langle \tilde{W},W^{*}\rangle }^{2}}$ of 5 random trials. (**a**)–(**c**) average recovery errors with θ=0; (**d**)–(**g**) average recovery errors with θ=0.1.

**Figure 4:**
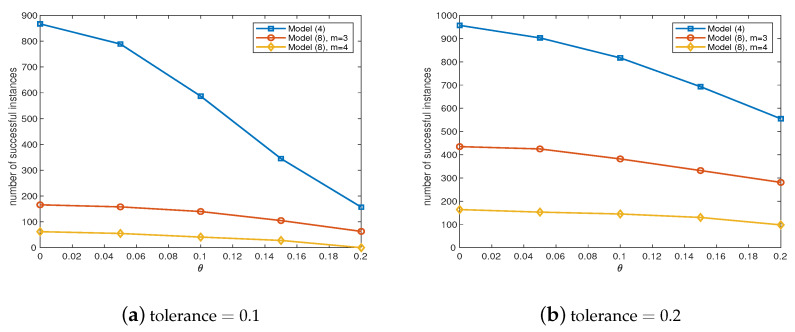
A comparison on the number of successfully recovered instances on the different level of noise. (**a**) $\sqrt{1-{\langle \tilde{W},W^{*}\rangle }^{2}}$ is less than 0.1; (**b**) $\sqrt{1-{\langle \tilde{W},W^{*}\rangle }^{2}}$ is less than 0.2.

In this subsection, we first verify the recovery property of our proposed model (8), which is a special case of (1) by fixing p=1. We first compare the distance between global minimizer of (8) and the ground-truth for DPCP problem. We first fix n=30 and randomly select $W^{*}\in R^{n}$. Then, we randomly generate $N_{1}$ inliers in the hyperplane whose normal vector is $W^{*}$. Besides, we randomly generate $N_{2}$ outliers in $R^{n}$ following Gaussian distribution. Additionally, the data is corrupted by Gaussian noise by adding $\frac{\theta }{\sqrt{n}}\cdot \mathrm{randn}\left(n,N\right)$ to *Y*. Then, we normalize each sample in *Y*. The range of $N_{1}$ is [10,500], whereas the range of $N_{2}$ is [10,3000]. We run each test problem for 5 instances. Moreover, in each instance, we run DPCP-PSGM to solve (4) and PenNMF to solve (8) with m=3 and 4, and get the solution $\tilde{W}$ for each model. We plot the principal angle between $\tilde{W}$ and $W^{*}$ in Figure 3. From Figure 3a,b we can conclude that (4) can tolerate $O(N_{1}^{2})$ outliers while achieve exact recovery, which coincides the theoretical results presented in [32]. For model (8), numerical experiments do not show the exact recovery ability of (8) for m=3 and 4. However, with some tolerance on the principal angle, we also observe that (4) can tolerate $O(N_{1}^{2})$ outliers. Moreover, we conclude that with m=3, (8) has better ability to recover the normal vector than m=4. As a result, in the rest of this subsection, we only test (8) with m=3. In addition, we analyze the number of successfully recovered instances, where the $\sqrt{1-{\langle \tilde{W},W^{*}\rangle }^{2}}$ is less than 0.1 or 0.2. The results are presented in Figure 4. From Figure 4, we can conclude that, with tolerance on the errors, the $\ell_{m}$-norm maximization model can successfully recover the normal vector. Moreover, in model (8), m=3 has better performance than m=4, which coincides with the numerical experiments in [18]. Therefore, when applying $\ell_{m}$-norm maximization model to solving the DPCP problems, we suggest to choose m=3 in (8).

In the rest of this subsection, we test the numerical performance of PenNMF on solving DPCP problem, which plays an important role in autonomous driving applications. DPCP is applied to recover the road-plane, which can be regarded as inliers, from the 3d point clouds in KITTA dataset [22], which is recorded from a moving platform while driving in and around Karlsruhe, Germany. This dataset consists of image data together with corresponding 3D points collected by a rotating 3D laser scanner [32]. Moreover, DPCP only uses the 3D point clouds with the objective of determining the 3D points that lie on the road plane (inliers) and those off that plane (outliers): Given a 3D point cloud of a road scene, the DPCP problem focuses on reconstructing an affine plane $\left\{x\in R^{3}|a^{⊤}x-b=0\right\}$ as a representation for the road. Equivalently, this task can be converted to a linear subspace learning problem by embedding the affine plane into the linear hyperplane $H\subseteq R^{4}$ with normal vector $\tilde{b}=\left[a,-b\right]$, through the mapping x→[x,1] [29]. We use the experimental set-up in [29,32] to further compare Equations (4) and (8), RANSAC, and other alternative methods in the task of 3D road plane detection in KITTA dataset. Each point cloud contains over $10^{5}$ samples with approximately 50% outliers. Besides, the samples are homogenized and normalized to unit $\ell_{2}$-norm.

We use 11 frames annotated in [29,32] from KITTA dataset. We compare DPCP-PSGM [29], DPCP-IRLS, and DPCP-d [31], which focus on solving the $\ell_{1}$-norm minimization model (4). Besides, we test RANSAC and $\ell_{2,1}$-RPCA [25]. Additionally, we test PenNMF and MSP/GPM on solving our proposed model (8), which is a special case of (1). For DPCP-PSGM, DPCP-d, DPCP-IRLS, and $\ell_{2,1}$-RPCA, all parameters are set by following the suggestions in [32].

**Figure 5:**
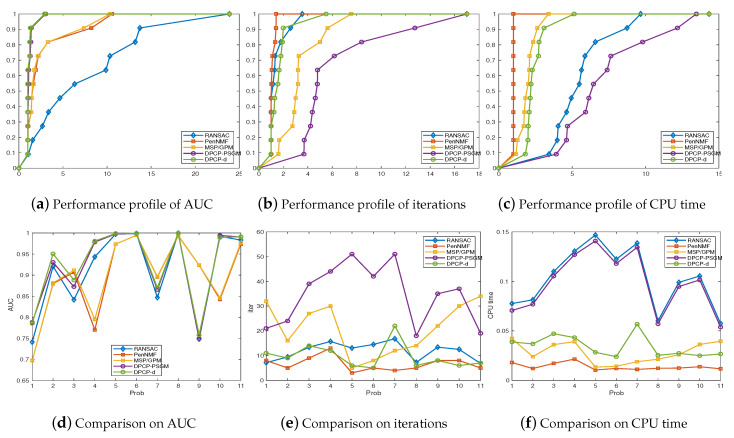
A comparison between PenNMF, MSP, DPCP-PSGM, DPCP-D, and Random Sampling and Consensus (RANSAC). (**a**)–(**c**) performance profile [41] of AUC, iterations and CPU time; (**d**)–(**f**) the numerical results of AUC, iterations and CPU time.

**Figure 6:**
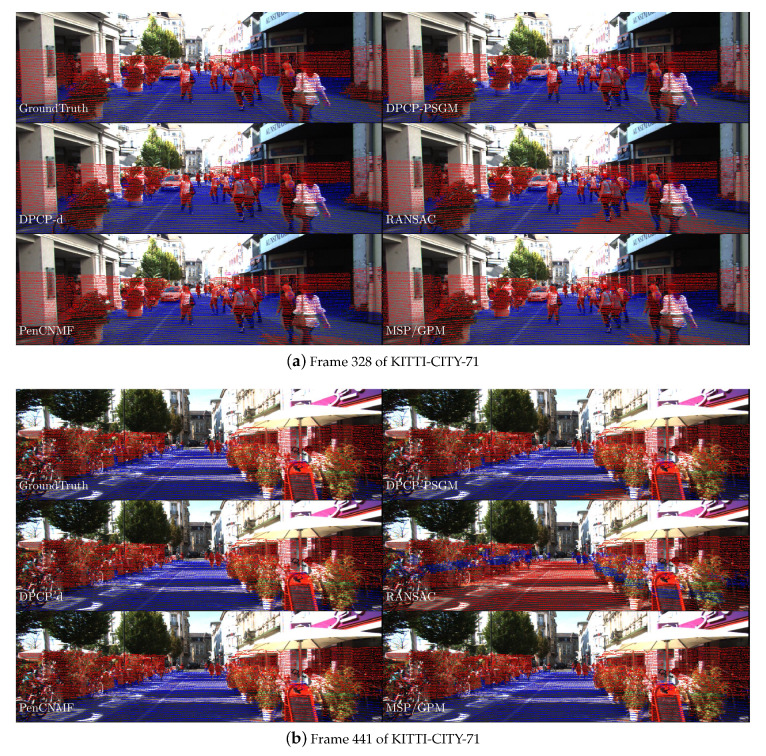
Illustrations to some results in our numerical tests, with inliers in blue and outliers in red. (**a**) Frame 328 from KITTI-CITY-71, N=121766; (**b**) Frame 441 from KITTI-CITY-71, N=119428. Inliers/outliers are detected by using a ground-truth thresholding on the distance to the hyperplane recovered by each compared method. The results are represented by projecting 3D point clouds onto the image.

Figure 5 illustrates the numerical performance of all the compared algorithms. We present the numerical results in Figure 5d–f. Moreover, we draw the performance profiles proposed by Dolan and Moré [41] in Figure 5a–c to present an illustrative comparison on the performance of all compared algorithms. The performance profiles can be regarded as distribution functions for a performance metric for benchmarking and comparing optimization algorithms. Besides, we draw the recovery results of frames 328 and 441 in KITTA-CITY-71, which is presented in Figure 6. Here the term “AUC” denotes the area under the AUC curve, and “iterations” denotes the total iterations taken by these compared algorithms. Besides, “Prob” in Figure 5d–f denotes the indexes of tested frames, which are presented in Table 1.

From Figure 5a, we can conclude that PenNMF and MSP/GPM successfully recover the hyperplanes with comparable accuracy. Moreover, in problems 3,7, and 9, PenNMF and MSP produce better classification accuracy than other approaches. Besides, in the aspect of CPU time, PenNMF and MSP cost much less time than other compared algorithms in most cases. Moreover, from Figure 5c, we can conclude that PenNMF takes less time than MSP as well as other compared algorithms in almost all the cases. As a result, we can conclude that our proposed model (1) is easy to be solved and PenNMF shows better efficiency than MSP in our test examples.

## 5. Conclusions

Sparse dictionary learning (SDL) and dual principal pursuit (DPCP) are two powerful tools in data science. In this paper, we formulate DPCP as a special case of the $\ell_{m}$-norm maximization on the Stiefel manifold proposed for SDL. Then, we propose a novel smooth unconstrained penalty model PenNM for the original optimization problem with orthogonality constraints. We show PenNM is an exact penalty function of (1) under mild assumptions. We develop an novel approximate gradient approach PenNMF for solving PenNM. The global convergence of PenNMF as well as its sublinear convergence rate are established. Numerical experiments illustrate that our proposed approach enjoys better performance than MSP/GPM [17,18] on various testing problems.

## Figures and Tables

**Table 1:** The testing instances and their corresponding frames in KITTA dataset.

Dataset	KITTI-CITY-71				KITTI-CITY-5					KITTI-CITY-48	
Frame id.	221	328	441	881	1	45	120	137	153	0	21
Test id.	1	2	3	4	5	6	7	8	9	10	11

## Abbreviations

The following abbreviations are used in this manuscript:

DPCP	dual principal pursuit
DPCP-d	Denoised-DPCP
DPCP-IRLS	Iteratively-Reweighted-Least-Squares algorithm
DPCP-PSGM	Projected subgradient-based algorithm for solving DPCP
GPM	gradient projection method
MSP	matching, stretching, and projection
PenNM	penalty model for $\ell_{m}$-norm maximization
PenNMF	first-order algorithm for solving our penalty model
RANSAC	Random Sampling and Consensus
SDL	Sparse dictionary learning

## Appendix A. Proof for Theorem 2 and Corollary 3

In this section, we present the proof for Theorem 2 and Corollary 3. As (6) is an unconstrained optimization problem, the upper-bound for ${∥\nabla h(W)∥}_{F}$ should be estimated. Before estimating the upper bounds of ${∥\nabla f(W)∥}_{F}$ and ${∥\nabla^{2}f\left(W\right)\left[W\left(W^{⊤}W-I_{p}\right)\right]∥}_{F}$, we first present two linear algebraic inequalities:

**Lemma** **A1.**
*For any $A,B\in R^{n\times N}$, ${∥A\circ B∥}_{F}\leq {∥A∥}_{F}{∥B∥}_{F}$.*

**Proof.** 

$$\begin{array}{c} {∥A\circ B∥}_{F}^{2}=\sum_{i}\sum_{j}A_{ij}^{2}B_{ij}^{2}\leq \left(\sum_{i}\sum_{j}A_{ij}^{2}\right)\left(\sum_{i}\sum_{j}B_{ij}^{2}\right)={∥A∥}_{F}^{2}{∥B∥}_{F}^{2}. \end{array}$$

 □

**Lemma** **A2.**
*For any $A\in R^{n\times N}$ and any m≥3, we have ${∥A^{\circ (m)}∥}_{F}\leq {∥A∥}_{F}^{m}$.*

**Proof.** This lemma directly follows the fact that

$${∥A^{\circ (m)}∥}_{F}^{2}=\sum_{i=1}^{n}\sum_{j=1}^{N}A_{i,j}^{2m}\leq {\left(\sum_{i=1}^{n}\sum_{j=1}^{N}A_{i,j}^{2}\right)}^{m}={∥A∥}_{F}^{2m}.$$

 □

Now, we present the upper bound estimation for ${∥\nabla f(W)∥}_{F}$ and ${∥\nabla^{2}f\left(W\right)\left[W\left(W^{⊤}W-I_{p}\right)\right]∥}_{F}$.

**Lemma** **A3.**
*For any $W\in R^{n\times p}$, ${∥\nabla f(W)∥}_{F}\leq {∥Y∥}_{F}^{m}{∥W∥}^{m-1}.$*

**Proof.** Due to the fact that $\nabla f\left(W\right)=Y\left[{\left(Y^{⊤}W\right)}^{\circ (m-1)}\circ \mathrm{sign}\left(Y^{⊤}W\right)\right]$,

$$\begin{array}{cc} {∥\nabla f(W)∥}_{F}\leq & {∥Y∥}_{F}{∥{\left(Y^{⊤}W\right)}^{\circ (m-1)}\circ \mathrm{sign}\left(Y^{⊤}W\right)∥}_{F}\\ = & {∥Y∥}_{F}{∥{\left(Y^{⊤}W\right)}^{\circ (m-1)}∥}_{F}\\ \leq & {∥Y∥}_{F}{∥Y^{⊤}W∥}_{F}^{m-1}\\ \leq & {∥Y∥}_{F}^{m}{∥W∥}^{m-1}. \end{array}$$

Here, the last inequality follows the fact that ${∥AB∥}_{F}\leq {∥A∥}_{F}∥B∥\leq {∥A∥}_{F}{∥B∥}_{F}$. □

**Lemma** **A4.**
*For any $W\in R^{n\times p}$,*

$${∥\nabla^{2}f\left(W\right)\left[W\left(W^{⊤}W-I_{p}\right)\right]∥}_{F}\leq \left(m-1\right){∥W∥}^{m-2}∥W(W^{⊤}W-I_{p})∥{∥Y∥}_{F}^{m}.$$

**Proof.** From the expression of $\nabla^{2}f\left(W\right)$ in Proposition 1,

$$\begin{array}{cc} \; & {∥\nabla^{2}f\left(W\right)\left[W\left(W^{⊤}W\right)\right]∥}_{F}\\ = & \left(m-1\right){∥Y\left[{\left(Y^{⊤}W\right)}^{\circ (m-2)}\circ \left(Y^{⊤}W\left(W^{⊤}W-I_{p}\right)\right)\right]∥}_{F}\\ \leq & \left(m-1\right){∥Y∥}_{F}{∥{\left(Y^{⊤}W\right)}^{\circ (m-2)}\circ \left(Y^{⊤}W\left(W^{⊤}W-I_{p}\right)\right)∥}_{F}\\ \leq & \left(m-1\right){∥Y∥}_{F}{∥{\left(Y^{⊤}W\right)}^{\circ (m-2)}∥}_{F}{∥Y^{⊤}W\left(W^{⊤}W-I_{p}\right)∥}_{F}\\ \leq & \left(m-1\right){∥W∥}^{m-1}∥W(W^{⊤}W-I_{p})∥{∥Y∥}_{F}^{m}. \end{array}$$

Here, the second inequality directly uses Lemma A1 and the last inequality follows Lemma A2. □

In the rest of this section, we consider the equivalence between (1) and (6). We first establish the relationships between the first-order stationary points of (6) and problem (1).

From the optimality condition of (6), we derive an important equality in Lemma A5.

**Lemma** **A5.**
*For any first-order stationary point $\tilde{W}$ of (6) and any symmetric matrix $T\in R^{p\times p}$ that satisfies $T{\tilde{W}}^{⊤}\tilde{W}={\tilde{W}}^{⊤}\tilde{W}T$, we have*

(A1) $$\begin{array}{cc} 0= & \mathrm{tr}\left(T\left({\tilde{W}}^{⊤}\tilde{W}-I_{p}\right)\left(\beta {\tilde{W}}^{⊤}\tilde{W}\left({\tilde{W}}^{⊤}\tilde{W}+I_{p}\right)-\frac{3}{2}\Lambda \left(\tilde{W}\right)\right)\right)\\ \; & -\frac{1}{2}\mathrm{tr}\left(T{\tilde{W}}^{⊤}\nabla^{2}f\left(\tilde{W}\right)\left[\tilde{W}\left({\tilde{W}}^{⊤}\tilde{W}-I_{p}\right)\right]\right). \end{array}$$

**Proof.** Suppose $\tilde{W}$ is a first-order stationary point of (6), by the first-order optimality condition, $\nabla h(\tilde{W})=0$. Then, for any symmetric matrix $T\in R^{p\times p}$ that satisfies $T{\tilde{W}}^{⊤}\tilde{W}={\tilde{W}}^{⊤}\tilde{W}T$, $\langle \tilde{W}T,\nabla h\left(\tilde{W}\right)\rangle =0$. As described in Proposition 1, ∇f(W) can be separated into three parts, we estimate their inner-product with $\tilde{W}T$ respectively. First,

$$\begin{array}{cc} \; & \langle \tilde{W}T,\nabla f\left(\tilde{W}\right)\left(\frac{3}{2}I_{p}-\frac{1}{2}{\tilde{W}}^{⊤}\tilde{W}\right)-\tilde{W}\Phi \left({\tilde{W}}^{⊤}\nabla f\left(\tilde{W}\right)\right)\rangle \\ = & \mathrm{tr}\left(T{\tilde{W}}^{⊤}\nabla f\left(\tilde{W}\right)\left(\frac{3}{2}I_{p}-\frac{1}{2}{\tilde{W}}^{⊤}\tilde{W}\right)-T{\tilde{W}}^{⊤}\tilde{W}\Lambda \left(\tilde{W}\right)\right)\\ = & \mathrm{tr}\left(T\left(\frac{3}{2}I_{p}-\frac{1}{2}{\tilde{W}}^{⊤}\tilde{W}\right)\Lambda \left(\tilde{W}\right)-T{\tilde{W}}^{⊤}\tilde{W}\Lambda \left(\tilde{W}\right)\right)\\ = & \frac{3}{2}\mathrm{tr}\left(T\left(I_{p}-{\tilde{W}}^{⊤}\tilde{W}\right)\Lambda \left(\tilde{W}\right)\right). \end{array}$$

Here, the second equality follows the fact that that $\mathrm{tr}\left(BC\right)=\mathrm{tr}\left(BCB\right)=0$ holds for any symmetric *B* and skew-symmetric *C*. Besides, the last inequality follows that $\left({\tilde{W}}^{⊤}\tilde{W}-I_{p}\right)T=T\left({\tilde{W}}^{⊤}\tilde{W}-I_{p}\right)$. As a result, we achieve the following equality:

$$\begin{array}{cc} \; & \langle \tilde{W}T,\nabla f\left(\tilde{W}\right)\left(\frac{3}{2}I_{p}-\frac{1}{2}{\tilde{W}}^{⊤}\tilde{W}\right)-\tilde{W}\Phi \left({\tilde{W}}^{⊤}\nabla f\left(\tilde{W}\right)\right)\rangle -\frac{1}{2}\langle \tilde{W}T,\nabla^{2}f\left(\tilde{W}\right)\left[\tilde{W}\left({\tilde{W}}^{⊤}\tilde{W}-I_{p}\right)\right]\rangle \\ = & -\frac{3}{2}\mathrm{tr}\left(T\left({\tilde{W}}^{⊤}\tilde{W}-I_{p}\right)\Lambda \left(\tilde{W}\right)\right)-\frac{1}{2}\langle T\tilde{W},\nabla^{2}f\left(\tilde{W}\right)\left[\tilde{W}\left({\tilde{W}}^{⊤}\tilde{W}-I_{p}\right)\right]\rangle . \end{array}$$

Additionally, we estimate their inner-product of $\tilde{W}T$ and $\beta \tilde{W}\left({\tilde{W}}^{⊤}\tilde{W}{\tilde{W}}^{⊤}\tilde{W}-I_{p}\right)$ and achieve the following equality

$$\begin{array}{cc} \; & \langle \tilde{W}T,\beta \left({\tilde{W}}^{⊤}\tilde{W}{\tilde{W}}^{⊤}\tilde{W}-I_{p}\right)\rangle \\ = & \langle \tilde{W}T,\beta \tilde{W}\left({\tilde{W}}^{⊤}\tilde{W}+I_{p}\right)\left({\tilde{W}}^{⊤}\tilde{W}-I_{p}\right)\rangle \\ = & \beta \mathrm{tr}\left(T{\tilde{W}}^{⊤}\tilde{W}\left({\tilde{W}}^{⊤}\tilde{W}+I_{p}\right)\left({\tilde{W}}^{⊤}\tilde{W}-I_{p}\right)\right). \end{array}$$

Based on the above two equations, multiplying $\left({\tilde{W}}^{⊤}\tilde{W}-I_{p}\right){\tilde{W}}^{⊤}$ on both sides of $0=\nabla h(\tilde{W})$ results in

(A2) $$\begin{array}{cc} 0= & \langle \tilde{W}T,\nabla h\left(\tilde{W}\right)\rangle \\ = & \mathrm{tr}\left(T\left({\tilde{W}}^{⊤}\tilde{W}-I_{p}\right)\left(\beta {\tilde{W}}^{⊤}\tilde{W}\left({\tilde{W}}^{⊤}\tilde{W}+I_{p}\right)-\frac{3}{2}\Lambda \left(\tilde{W}\right)\right)\right)\\ \; & -\frac{1}{2}\mathrm{tr}\left(T{\tilde{W}}^{⊤}\nabla^{2}f\left(\tilde{W}\right)\left[\tilde{W}\left({\tilde{W}}^{⊤}\tilde{W}-I_{p}\right)\right]\right), \end{array}$$

and thus we complete the proof. □

Then based on the equality in Lemma A5, the following proposition shows that all first-order stationary point of (6) is uniformly bounded.

**Proposition** **A6.**
*For any first-order stationary point $\tilde{W}$ of (6), suppose $\beta \geq \left(4m+8\right){∥Y∥}_{F}^{m}$, then ${∥\tilde{W}∥}^{2}\leq 1+\frac{\left(m+2\right){∥Y∥}_{F}^{m}}{\beta }$.*

**Proof.** Let *u* denotes the top eigenvector of ${\tilde{W}}^{⊤}\tilde{W}$, i.e. ${\tilde{W}}^{⊤}\tilde{W}u=∥{\tilde{W}}^{⊤}\tilde{W}∥u$. Suppose $\tilde{W}$ is a first-order stationary point that satisfies ${∥\tilde{W}∥}^{2}>1+\frac{\left(m+2\right){∥Y∥}_{F}^{m}}{\beta }$. By Lemma A5 we first have

(A3) $$\begin{array}{cc} 0= & \langle uu^{⊤}\tilde{W},\nabla f\left(\tilde{W}\right)\rangle \\ = & \mathrm{tr}\left(\beta uu^{⊤}\left({\tilde{W}}^{⊤}\tilde{W}-I_{p}\right){\tilde{W}}^{⊤}\tilde{W}\left({\tilde{W}}^{⊤}\tilde{W}+I_{p}\right)\right)-\frac{3}{2}\mathrm{tr}\left(uu^{⊤}\left({\tilde{W}}^{⊤}\tilde{W}-I_{p}\right)\Lambda \left(\tilde{W}\right)\right)\\ & -\frac{1}{2}\mathrm{tr}\left(uu^{⊤}{\tilde{W}}^{⊤}\nabla^{2}f\left(\tilde{W}\right)\left[\tilde{W}\left({\tilde{W}}^{⊤}\tilde{W}-I_{p}\right)\right]\right)\\ \geq & \beta \left({∥\tilde{W}∥}^{6}-{∥\tilde{W}∥}^{2}\right)-\frac{3}{2}{∥\tilde{W}∥}^{2}u^{⊤}\Lambda \left(\tilde{W}\right)u-\frac{m-1}{2}{∥Y∥}_{F}^{m}{∥\tilde{W}∥}^{m+2}\\ \geq & \beta \left({∥\tilde{W}∥}^{6}-{∥\tilde{W}∥}^{2}\right)-\frac{\left(m+2\right){∥Y∥}_{F}^{m}{∥\tilde{W}∥}^{m+2}}{2}\\ \geq & \left(\beta -\frac{\left(m+2\right){∥Y∥}_{F}^{m}}{2}\right){∥\tilde{W}∥}^{6}-\beta {∥\tilde{W}∥}^{4}\\ = & {∥\tilde{W}∥}^{4}\left[\left(\beta -\frac{\left(m+2\right){∥Y∥}_{F}^{m}}{2}\right){∥\tilde{W}∥}^{2}-\beta \right]\\ > & 0, \end{array}$$

which leads to the contradictory and shows that ${∥\tilde{W}∥}^{2}\leq 1+\frac{\left(m+2\right){∥Y∥}_{F}^{m}}{\beta }$. Here, the second equality directly follows Lemma A5. The first inequality uses Lemma A4 and the fact that $∥\tilde{W}\left({\tilde{W}}^{⊤}\tilde{W}-I_{p}\right)∥\leq {∥\tilde{W}∥}^{3}$. Besides, the second inequality follows the fact that ${\tilde{W}}^{⊤}\tilde{W}⪰{∥\tilde{W}∥}^{2}uu^{⊤}$. The second inequality uses the fact that $u^{⊤}\Lambda \left(\tilde{W}\right)u\leq {∥\Lambda (\tilde{W})∥}_{F}\leq {∥{\tilde{W}}^{⊤}\nabla f\left(\tilde{W}\right)∥}_{F}\leq {∥\tilde{W}∥}_{2}{∥\nabla f(\tilde{W})∥}_{F}$. The fourth inequality uses the fact that $\mathrm{tr}\left({\tilde{W}}^{⊤}\tilde{W}-I_{p}\right)\leq {∥\tilde{W}∥}_{F}^{2}$, and the last inequality follows the fact that ${∥\tilde{W}∥}^{2}-1\geq \frac{\left(m+2\right){∥Y∥}_{F}^{m}}{\beta }$. □

Combine Lemma A5 and Proposition A6, we restate Theorem 2 as Theorem A7 and achieve the equivalence between (1) and (6).

**Theorem** **A7.**
*Suppose $\beta \geq \left(4m+8\right){∥Y∥}_{F}^{m}$, and $\tilde{W}$ is a first-order stationary point of (6), then either ${\tilde{W}}^{⊤}\tilde{W}=I_{p}$ holds, which further implies that $\tilde{W}$ is a first-order stationary point of problem (1), or the inequality $\sigma_{\min}\left({\tilde{W}}^{⊤}\tilde{W}\right)\leq \frac{\left(2m+4\right){∥Y∥}_{F}^{m}}{\beta }$ holds.*

**Proof.** When $\beta \geq 4\left(m+2\right){∥Y∥}_{F}^{m}$, any first-order stationary point $\tilde{W}$ of (6) satisfies that ${∥\tilde{W}∥}^{2}\leq 2$. Suppose $\tilde{W}$ satisfies ${\tilde{W}}^{⊤}\tilde{W}⪰\frac{\left(2m+4\right){∥Y∥}_{F}^{m}}{\beta }I_{p}$, then $\frac{\beta }{2}{\tilde{W}}^{⊤}\tilde{W}\left({\tilde{W}}^{⊤}\tilde{W}+I_{p}\right)⪰\left(m+2\right){∥\tilde{W}∥}^{m}{∥Y∥}_{F}^{m}\cdot I_{p}$. Then from Lemma A5, we have

$$\begin{array}{cc} 0= & \mathrm{tr}\left({\left({\tilde{W}}^{⊤}\tilde{W}-I_{p}\right)}^{2}\left(\beta {\tilde{W}}^{⊤}\tilde{W}\left({\tilde{W}}^{⊤}\tilde{W}+I_{p}\right)-\frac{3}{2}\Lambda \left(\tilde{W}\right)\right)\right)\\ & -\frac{1}{2}\mathrm{tr}\left(\left({\tilde{W}}^{⊤}\tilde{W}-I_{p}\right){\tilde{W}}^{⊤}\nabla^{2}f\left(\tilde{W}\right)\left[\tilde{W}\left({\tilde{W}}^{⊤}\tilde{W}-I_{p}\right)\right]\right)\\ \geq & \mathrm{tr}\left({\left({\tilde{W}}^{⊤}\tilde{W}-I_{p}\right)}^{2}\left(\beta {\tilde{W}}^{⊤}\tilde{W}\left({\tilde{W}}^{⊤}\tilde{W}+I_{p}\right)-\frac{m+2}{2}{∥W∥}^{m}{∥Y∥}_{F}^{m}\cdot I_{p}\right)\right)\\ \geq & \mathrm{tr}\left(\beta {\left({\tilde{W}}^{⊤}\tilde{W}-I_{p}\right)}^{2}{\tilde{W}}^{⊤}\tilde{W}\left({\tilde{W}}^{⊤}\tilde{W}+I_{p}\right)\right)\\ \geq & 0, \end{array}$$

showing that $\mathrm{tr}\left(\beta {\left({\tilde{W}}^{⊤}\tilde{W}-I_{p}\right)}^{2}{\tilde{W}}^{⊤}\tilde{W}\left({\tilde{W}}^{⊤}\tilde{W}+I_{p}\right)\right)=0$. Then by the positive-definiteness of ${\tilde{W}}^{⊤}\tilde{W}$, we can conclude that ${\tilde{W}}^{⊤}\tilde{W}-I_{p}=0$. As a result, we have that either ${\tilde{W}}^{⊤}\tilde{W}-I_{p}=0$ or $\sigma_{\min}\left({\tilde{W}}^{⊤}\tilde{W}\right)\leq \frac{\left(2m+4\right){∥Y∥}_{F}^{m}}{\beta }$, and completes the proof. □

**Corollary** **A8.**
*Suppose p=1 in (1), $\beta \geq \left(4m+8\right){∥Y∥}_{F}^{m}$, and $\tilde{W}$ is a first-order stationary point of (6), then either ${\tilde{W}}^{⊤}\tilde{W}=I_{p}$ holds, which further implies that $\tilde{W}$ is a first-order stationary point of problem (1), or $\tilde{W}=0$.*

**Proof.** By the same routine of Theorem 2, when $\beta \geq 4\left(m+2\right){∥Y∥}_{F}^{m}$, any first-order stationary point $\tilde{W}$ of (6) satisfies that ${∥\tilde{W}∥}^{2}\leq 2$. Then, following the same proof routine in Lemma A5 and Theorem 2, we have

$$\begin{array}{cc} 0\geq & \mathrm{tr}\left({\left({\tilde{W}}^{⊤}\tilde{W}-I_{p}\right)}^{2}\left(\beta {\tilde{W}}^{⊤}\tilde{W}\left({\tilde{W}}^{⊤}\tilde{W}+I_{p}\right)-\frac{m+2}{2}{∥W∥}^{m}{∥Y∥}_{F}^{m}\cdot I_{p}\right)\right)\\ = & {\left({∥\tilde{W}∥}^{2}-1\right)}^{2}\left(\beta {∥\tilde{W}∥}^{2}\left({∥\tilde{W}∥}^{2}+I_{p}\right)-\frac{m+2}{2}{∥W∥}^{m}{∥Y∥}_{F}^{m}\right)\\ \geq & 0. \end{array}$$

When $\beta \geq \frac{4m+8}{2}{∥Y∥}_{F}$, we have

(A4) $$\frac{\beta }{2}{∥W∥}_{F}^{4}+\frac{\beta }{2}{∥W∥}_{F}^{2}>\frac{m+2}{2}{∥Y∥}_{F}{∥\tilde{W}∥}_{2}^{m}$$

holds for any $W\in R^{n}\setminus 0$. Then, for any $\tilde{W}\neq 0$, we can conclude that

$$\left(\beta {∥\tilde{W}∥}^{2}\left({∥\tilde{W}∥}^{2}+I_{p}\right)-\frac{m+2}{2}{∥W∥}^{m}{∥Y∥}_{F}^{m}\right)>0,$$

and thus ${∥\tilde{W}∥}^{2}-1=0$. As a result, from (A1), when $\tilde{W}$ is a first-order stationary point of (6), either $\tilde{W}=0$ or ${\tilde{W}}^{⊤}\tilde{W}-I_{p}=0$. □

## Appendix B. Proof for Theorem 4

In this section, we present the main body of the proof for Theorem 4. To show the convergence of PenNMF, we first present some preliminary lemmas. Then, we show that the updating direction $D(W^{k})$ is a descending direction and thus $h\left(W^{k+1}\right)\leq h\left(W^{k}\right)$, as illustrated in Lemma A12. Together with Lemma A10, we show that the sequence is restricted in the neighborhood of the constraints, and we achieve the global convergence property of PenNMF in Theorem 4. We first estimate the upper-bound of the term $\left|f\left(W\right)-\frac{1}{2}\langle W^{⊤}W-I_{p},\Lambda \left(W\right)\rangle \right|$ in h(W).

**Lemma** **A9.**
*For any $W\in R^{n\times p}$,*

$$\left|f\left(W\right)-\frac{1}{2}\langle W^{⊤}W-I_{p},\Lambda \left(W\right)\rangle \right|\leq \frac{m}{2}{∥Y∥}_{F}^{m}\max\left\{{∥W∥}^{2}+1,2\right\}{∥W∥}^{m}.$$

**Proof.** We first estimate the upper-bound for |f(W)|, which can be achieved by

$$\left|f\left(W\right)\right|=\frac{1}{m}{∥W^{⊤}Y∥}_{m}^{m}\leq \frac{1}{m}{∥W^{⊤}Y∥}_{F}^{m}\leq \frac{1}{m}{∥Y∥}_{F}^{m}{∥W∥}^{m}.$$

Besides, from Lemma A3, we have

$$\begin{array}{cc} \; & \left|\frac{1}{2}\langle W^{⊤}W-I_{p},\Lambda \left(W\right)\rangle \right|=\frac{1}{2}\left|\langle W\left(W^{⊤}W-I_{p}\right),\nabla f\left(W\right)\rangle \right|\\ \leq & \frac{1}{2}{∥W(W^{⊤}W-I_{p})∥}_{F}{∥\nabla f(W)∥}_{F}\\ \leq & \frac{m}{2}{∥W(W^{⊤}W-I_{p})∥}_{2}{∥Y∥}_{F}^{m}{∥W∥}^{m-1}\\ \leq & \frac{m}{2}{∥Y∥}_{F}^{m}\max\left\{1,{∥W∥}^{2}\right\}{∥W∥}^{m}. \end{array}$$

Combine the above two equations, we achieve

$$|f\left(W\right)-\frac{1}{2}\langle W^{⊤}W-I_{p},\Lambda \left(W\right)\rangle |\leq \frac{m}{2}{∥Y∥}_{F}^{m}\max\left\{{∥W∥}^{2}+1,2\right\}{∥W∥}^{m},$$

and complete the proof. □

We then show that the penalty term $\psi \left(W\right):=\frac{1}{6}{∥W∥}_{F}^{6}-\frac{1}{2}{∥W∥}_{F}^{2}$ builds a barrier around $S_{n,p}$, i.e., those points that are sufficiently far from $S_{n,p}$ have higher functional value than those points that are close to $S_{n,p}$.

**Lemma** **A10.**
*Suppose for any $\delta \in \left(0,\frac{1}{3}\right]$ and $\beta \geq \max\{228m{∥Y∥}_{F}^{m},\frac{32m}{\delta }{∥Y∥}_{F}^{m}\}$, we have*

(A5) $$\max_{{∥W^{⊤}W-I_{p}∥}_{F}^{2}\leq \frac{\delta }{8}}h\left(W\right)\leq \min_{{∥W^{⊤}W-I_{p}∥}_{F}^{2}\geq \delta }h\left(W\right).$$

**Proof.** Let $\psi \left(W\right):=\frac{1}{6}\left({∥W∥}^{6}-{∥W∥}^{2}\right)$. For any $W_{1}$ satisfies $\psi (W_{1})\leq \delta$ and $W_{2}$ satisfies ${∥W_{2}∥}^{2}\leq 2$ and $\psi (W_{2})\geq 2\delta$, then

(A6) $$\begin{array}{cc} \; & h\left(W_{2}\right)-h\left(W_{1}\right)\\ \geq & \beta \delta -|\nabla f\left(W_{1}\right)|-\frac{1}{2}\langle W_{1}^{⊤}W_{1}-I_{p},\Lambda \left(W_{1}\right)\rangle |-|\nabla f\left(W_{2}\right)\left|-\frac{1}{2}\langle W_{2}^{⊤}W_{2}-I_{p},\Lambda \left(W_{2}\right)\rangle \right|\\ \geq & \beta \delta -m{∥Y∥}_{F}^{m}{\left(1+\delta \right)}^{m+2}-m{∥Y∥}_{F}^{m}2^{m+2}\\ \geq & \beta \delta -19m{∥Y∥}_{F}^{m}\\ \geq & 0. \end{array}$$

Here, the second inequality uses the fact that ${∥W_{1}∥}_{2}^{2}\leq 1+{∥W_{1}^{⊤}W_{1}-I_{p}∥}_{F}\leq 1+\delta$, and ${∥W_{2}∥}^{2}\leq 2$. Moreover, when ${∥W_{2}∥}_{F}^{2}\geq 2$, we have $\psi \left(W_{2}\right)\geq \frac{\beta }{6}{∥\tilde{W}∥}^{6}$. Then, $\frac{1}{2}\psi \left(W_{2}\right)\geq \frac{1}{3}\beta \geq \delta \beta \geq \psi \left(W_{1}\right)$.

(A7) $$\begin{array}{cc} h\left(W_{2}\right)-h\left(W_{1}\right)\geq & -m{∥Y∥}_{F}^{m}{\left(1+\delta \right)}^{m+2}+\psi \left(W_{2}\right)-16m{∥Y∥}_{F}^{m}{∥W∥}^{m+2}-\delta \beta \\ \geq & -19m{∥Y∥}_{F}^{m}{∥W_{2}∥}^{m+2}+\frac{\beta }{12}{∥\tilde{W}∥}^{6}\\ \geq & 0. \end{array}$$

Here, the second inequality follows the fact that ${\left(1+\delta \right)}^{m}\leq {\left(1+\delta \right)}^{4}<3$. Besides, as ${∥W^{⊤}W-I_{p}∥}_{F}^{2}\leq \delta$ implies

(A8) $$\frac{1}{6}{∥W∥}_{F}^{6}-\frac{1}{2}{∥W∥}_{F}^{2}+\frac{p}{3}=\frac{1}{6}\mathrm{tr}\left[{\left(W^{⊤}W-I_{p}\right)}^{2}\left(W^{⊤}W+2I_{p}\right)\right].$$

As a result, ψ(W)≤3δ implies ${∥W^{⊤}W-I_{p}∥}_{F}\leq \delta$. Besides, ${∥W^{⊤}W-I_{p}∥}_{F}^{2}\leq \delta$ implies $\psi \left(W\right)\leq \frac{2}{3}\delta$. Therefore,

(A9) $$\max_{{∥W^{⊤}W-I_{p}∥}_{F}^{2}\leq \frac{\delta }{8}}h\left(W\right)\leq \min_{{∥W^{⊤}W-I_{p}∥}_{F}^{2}\geq \delta }h\left(W\right).$$

 □

Lemma A10 shows that the smooth penalty term builds a barrier around $S_{n,p}$. Moreover, we characterize the relations between ${∥D(W)∥}_{F}$ and ${∥W^{⊤}W-I_{p}∥}_{F}$ in the following lemma.

**Lemma** **A11.**
*Suppose $\delta \in \left(0,\frac{1}{3}\right]$, set $\left|\right|W^{⊤}W-I_{p}{\left|\right|}_{F}^{2}\leq \frac{1}{8}\delta$, and $\beta \geq \left(4m+8\right){∥Y∥}_{F}^{m}$. Then,*

(A10) $$\begin{array}{c} {∥D(W)∥}_{F}\geq \frac{\sqrt{3}\beta }{6}\cdot {∥W^{⊤}W-I_{p}∥}_{F}, \end{array}$$

*where $D\left(W\right):=\nabla f\left(W\right)-W\Phi \left(W^{⊤}\nabla f\left(W\right)\right)+\beta W\left(W^{⊤}WW^{⊤}W-I_{p}\right)$.*

**Proof.** First, we present two linear algebra relationships: The first is the inequality ${\left||A|\right|}_{F}\geq {∥\frac{A+A^{⊤}}{2}∥}_{F}$ holds for any square matrix *A*, which is quite obvious and the proof is omitted. The second is the equality $||AB+{BA||}_{F}={2||AB||}_{F}$ holds for any symmetric matrices *A* and *B*, which results from the fact $||AB+{BA||}_{F}^{2}={2||AB||}_{F}^{2}+2\mathrm{tr}\left(ABAB\right)={2||AB||}_{F}^{2}+2\mathrm{tr}\left(A^{\frac{1}{2}}BA^{\frac{1}{2}}A^{\frac{1}{2}}BA^{\frac{1}{2}}\right)={4||AB||}_{F}^{2}$. It follows from the above facts that

$$\begin{array}{cc} \; & {∥W^{⊤}D\left(W\right)∥}_{F}\geq \frac{1}{2}{∥W^{⊤}D\left(W\right)+D{\left(W\right)}^{⊤}W∥}_{F}\\ = & \frac{1}{2}{∥\left(\beta W^{⊤}W\left(W^{⊤}W+I_{p}\right)-\Lambda \left(W\right)\right)\left(W^{⊤}W-I_{p}\right)+\left(W^{⊤}W-I_{p}\right)\left(\beta W^{⊤}W\left(W^{⊤}W+I_{p}\right)-\Lambda \left(W\right)\right)∥}_{F}\\ \geq & \frac{\beta }{3}\cdot \left|\right|W^{⊤}W-I_{p}{\left|\right|}_{F}, \end{array}$$

where the last equality uses the fact that $\sigma_{\min}\left(W^{⊤}W\left(W^{⊤}W+I_{p}\right)\right)\geq \sigma_{\min}\left(W^{⊤}W\right)\geq 1-\delta \geq \frac{2}{3}$. Together with the facts that ${∥W^{⊤}D\left(W\right)∥}_{F}\leq {∥W∥}_{2}{∥D(W)∥}_{F}$ and $\sigma_{\max}\left(W^{⊤}W\right)\leq 1+\delta \leq \frac{4}{3}$, we have

$$\begin{array}{cc} {∥D(W)∥}_{F}\geq & \frac{1}{{∥W∥}_{F}}{∥W^{⊤}D\left(W\right)∥}_{F}\geq \frac{\sqrt{3}}{2}{∥W^{⊤}D\left(W\right)∥}_{F}\\ \geq & \frac{\sqrt{3}\beta }{6}{∥W^{⊤}W-I_{p}∥}_{F}. \end{array}$$

 □

Let $M_{1}:=\sup\frac{{∥\nabla h\left(W_{1}\right)-\nabla h\left(W_{2}\right)∥}_{F}}{{∥W_{1}-W_{2}∥}_{F}}$, then we have that the following illustrating that PenNMF generates a descending sequence $\left\{W_{k}\right\}$.

**Lemma** **A12.**
*Suppose $\delta \in \left(0,\frac{1}{3}\right]$ and $\beta \geq \max\left\{228m{∥Y∥}_{F}^{m},\frac{32}{\delta }{∥Y∥}_{F}^{m}\right\}$. Let $\left\{W^{k}\right\}$ be the iterate sequence generated by PenNMF, starting from any initial point $W^{0}$ satisfying $\left|\right|{W^{0}}^{⊤}W^{0}-I_{p}{\left|\right|}_{F}^{2}\leq \frac{1}{8}\delta$, and the stepsize $\eta_{k}\in \left[\frac{1}{2}\bar{\eta },\bar{\eta }\right]$, where $\bar{\eta }\leq \frac{1}{2M_{1}}$. Then, it holds that*

(A11) $$\begin{array}{c} h\left(W^{k+1}\right)\leq h\left(W^{k}\right)-\frac{\bar{\eta }}{4}{∥D(W^{k})∥}_{F}^{2} \end{array}$$

*for any k=0,1,⋯.*

**Proof.** By the explicit expression of $\nabla h(W^{k})$, we first have

(A12) $$\begin{array}{cc} \; & {∥\nabla h\left(W^{k}\right)-D\left(W^{k}\right)∥}_{F}\\ = & {∥\frac{1}{2}\nabla f\left(W^{k}\right)\left({W^{k}}^{⊤}W^{k}-I_{p}\right)+\frac{1}{2}\nabla^{2}f\left(W^{k}\right)\left[W^{k}\left({W^{k}}^{⊤}W^{k}-I_{p}\right)\right]∥}_{F}\\ \leq & \frac{1}{2}\left[{∥\nabla f\left(W^{k}\right)\left({W^{k}}^{⊤}W^{k}-I_{p}\right)∥}_{F}+{∥\nabla^{2}f\left(W^{k}\right)\left[W^{k}\left({W^{k}}^{⊤}W^{k}-I_{p}\right)\right]∥}_{F}\right]\\ \leq & \frac{m}{2}{∥Y∥}_{F}^{m}{∥W^{k}∥}^{m-1}{∥{W^{k}}^{⊤}W^{k}-I_{p}∥}_{F}\\ \leq & 2m{∥Y∥}_{F}^{m}{∥{W^{k}}^{⊤}W^{k}-I_{p}∥}_{F}. \end{array}$$

Here, the first inequality follows Lemma A3 and Lemma A4. Besides, by the definition of $M_{1}$, we have

(A13) $$h\left(W^{k+1}\right)\leq h\left(W^{k}\right)+\langle W^{k+1}-W^{k},\nabla h\left(W^{k}\right)\rangle +\frac{M_{1}}{2}{∥W^{k+1}-W^{k}∥}_{F}^{2}.$$

Suppose ${∥{W^{k}}^{⊤}W^{k}-I_{p}∥}_{F}^{2}\leq \delta$, then by Lemma A11 we can conclude that

(A14) $${∥\nabla h\left(W^{k}\right)-D\left(W^{k}\right)∥}_{F}\leq 2m{∥Y∥}_{F}^{m}{∥{W^{k}}^{⊤}W^{k}-I_{p}∥}_{F}\leq \frac{4\sqrt{3}m{∥Y∥}_{F}^{m}}{\beta }{∥D(W^{k})∥}_{F}.$$

Substitute $W^{k+1}-W^{k}=-\eta D\left(W^{k}\right)$ and (A14) into (A13), we have

(A15) $$\begin{array}{cc} \; & h\left(W^{k+1}\right)-h\left(W^{k}\right)\\ \leq & \langle W^{k+1}-W^{k},\nabla h\left(W^{k}\right)\rangle +\frac{M_{1}}{2}{∥W^{k+1}-W^{k}∥}_{F}^{2}\\ \leq & -\eta^{k}\langle D\left(W^{k}\right),D\left(W^{k}\right)\rangle +\frac{M_{1}}{2}{∥\eta^{k}D\left(W^{k}\right)∥}_{F}^{2}+\left|\langle \nabla h\left(W^{k}\right)-D\left(W^{k}\right),D\left(W^{k}\right)\rangle \right|\\ \leq & \left(-\eta^{k}+\frac{4\sqrt{3}m{∥Y∥}_{F}^{m}}{\beta }\eta^{k}+\frac{M_{1}}{2}{\left(\eta^{k}\right)}^{2}\right){∥D(W^{k})∥}_{F}^{2}\\ \leq & -\frac{\eta^{k}}{2}{∥D(W^{k})∥}_{F}^{2}\leq -\frac{\bar{\eta }}{4}{∥D(W^{k})∥}_{F}^{2}. \end{array}$$

Then by Lemma A10, as $h\left(W^{k+1}\right)\leq h\left(W^{k}\right)$, we can conclude that ${∥{W^{k+1}}^{⊤}W^{k+1}-I_{p}∥}_{F}^{2}\leq \delta$. Then, by induction we can conclude that ${∥{W^{k}}^{⊤}W^{k}-I_{p}∥}_{F}^{2}\leq \delta$ holds for k=1,2,3,⋯. Then, by (A15) again we conclude that

(A16) $$\begin{array}{c} h\left(W^{k+1}\right)\leq h\left(W^{k}\right)-\frac{\bar{\eta }}{4}{∥D(W^{k})∥}_{F}^{2} \end{array}$$

holds for k=1,2,3,⋯ and completes our proof. □

The following lemma shows that when our algorithm stops at $\tilde{W}$, then $\tilde{W}$ is a first-order stationary point of (1).

**Lemma** **A13.**
*Suppose $\delta \in \left(0,\frac{1}{3}\right]$ and $\beta \geq \max\left\{228m{∥Y∥}_{F}^{m},\frac{32}{\delta }{∥Y∥}_{F}^{m}\right\}$. For any $\tilde{W}$ satisfying ${∥{\tilde{W}}^{⊤}\tilde{W}-I_{p}∥}_{F}^{2}\leq \delta$ and $D(\tilde{W})=0$, we have that $\tilde{W}$ is a first-order stationary point of (1).*

**Proof.** Suppose $D(\tilde{W})=0$, then by the same proof routine in Theorem 2, we consider the inner-product of $D(\tilde{W})$ and $\tilde{W}\left({\tilde{W}}^{⊤}\tilde{W}-I_{p}\right)$:

$$\begin{array}{cc} 0= & \langle D\left(\tilde{W}\right),\tilde{W}\left({\tilde{W}}^{⊤}\tilde{W}-I_{p}\right)\rangle \\ = & \langle \nabla f\left(\tilde{W}\right)-\tilde{W}\Lambda \left(\tilde{W}\right)+\beta \tilde{W}\left({\tilde{W}}^{⊤}\tilde{W}{\tilde{W}}^{⊤}\tilde{W}-I_{p}\right),\tilde{W}\left({\tilde{W}}^{⊤}\tilde{W}-I_{p}\right)\rangle \\ = & \mathrm{tr}\left(\left({\tilde{W}}^{⊤}\tilde{W}-I_{p}\right){\tilde{W}}^{⊤}\nabla f\left(\tilde{W}\right)-\left({\tilde{W}}^{⊤}\tilde{W}-I_{p}\right){\tilde{W}}^{⊤}\tilde{W}\Lambda \left(\tilde{W}\right)+\beta {\tilde{W}}^{⊤}\tilde{W}\left({\tilde{W}}^{⊤}\tilde{W}+I_{p}\right){\left({\tilde{W}}^{⊤}\tilde{W}-I_{p}\right)}^{2}\right)\\ = & \mathrm{tr}\left({\left({\tilde{W}}^{⊤}\tilde{W}-I_{p}\right)}^{2}(\beta {\tilde{W}}^{⊤}\tilde{W}\left({\tilde{W}}^{⊤}\tilde{W}+I_{p}\right)-\Lambda \left(\tilde{W}\right)\right)\\ \geq & \mathrm{tr}\left({\left({\tilde{W}}^{⊤}\tilde{W}-I_{p}\right)}^{2}(\beta {\tilde{W}}^{⊤}\tilde{W}\left({\tilde{W}}^{⊤}\tilde{W}+I_{p}\right)-\left(4m+8\right){∥Y∥}_{F}^{m}\cdot I_{p}\right)\\ \geq & \frac{\beta }{2}\mathrm{tr}\left({\left({\tilde{W}}^{⊤}\tilde{W}-I_{p}\right)}^{2}\right)\geq 0. \end{array}$$

Here, the fourth equation follows the definition of $\Lambda \left(\tilde{W}\right):=\Phi \left({\tilde{W}}^{⊤}\nabla f\left(\tilde{W}\right)\right)$ and the first inequality uses the fact that $∥\tilde{W}∥\leq 2$, then together with Lemma A3, we can conclude that ${∥\Lambda (\tilde{W})∥}_{2}⪯\left(4m+8\right){∥Y∥}_{F}^{m}\cdot I_{p}$. Besides, the last inequality uses the fact that $\frac{\beta }{2}{\tilde{W}}^{⊤}\tilde{W}\left({\tilde{W}}^{⊤}\tilde{W}+I_{p}\right)⪰\left(4m+8\right){∥Y∥}_{F}^{m}\cdot I_{p}$. Then we can conclude that that ${\tilde{W}}^{⊤}\tilde{W}=I_{p}$. By the definition of $\nabla h(\tilde{W})$, we have

(A17) $$\nabla h\left(\tilde{W}\right)-D\left(\tilde{W}\right)=-\frac{1}{2}\nabla f\left(\tilde{W}\right)\left({\tilde{W}}^{⊤}\tilde{W}-I_{p}\right)-\frac{1}{2}\nabla^{2}f\left(\tilde{W}\right)\left[\tilde{W}\left({\tilde{W}}^{⊤}\tilde{W}-I_{p}\right)\right]=0,$$

showing that $\nabla f(\tilde{W})=0$. Together with Theorem 2 we can conclude that $\tilde{W}$ is a first-order stationary point of (1). □

Based on the Lemmas A10–A13, we restate Theorem 4 as Theorem A14 and show the global convergence property of PenNMF in the following theorem.

**Theorem** **A14.**
*Suppose $\delta \in \left(0,\frac{1}{3}\right]$ and $\beta \geq \max\left\{228m{∥Y∥}_{F}^{m},\frac{32}{\delta }{∥Y∥}_{F}^{m}\right\}$. Let $\left\{W^{k}\right\}$ be the iterate sequence generated by PenNMF, starting from any initial point $W^{0}$ satisfying $\left|\right|{W^{0}}^{⊤}W^{0}-I_{p}{\left|\right|}_{F}^{2}\leq \frac{1}{8}\delta$, and the stepsize $\eta_{k}\in \left[\frac{1}{2}\bar{\eta },\bar{\eta }\right]$, where $\bar{\eta }\leq \frac{1}{2M_{1}}$. Then, $W^{k}$ weakly converges to a first-order stationary point of (1). Moreover, for any k=1,2,⋯, the convergence rate of PenNMF can be estimated by*

(A18) $$\min_{0\leq i\leq k}{∥D(W^{i})∥}_{F}\leq \sqrt{\frac{8m{∥Y∥}_{F}^{m}+2\beta \delta }{\bar{\eta }\left(k+1\right)}}.$$

**Proof.** By Lemma A12, it holds that

$$h\left(W^{k+1}\right)\leq h\left(W^{k}\right)-\frac{\bar{\eta }}{4}{∥D(W^{k})∥}_{F}^{2}.$$

If $W^{*}$ is a cluster point of $\left\{W^{k}\right\}$, we have $W^{k+1}-W^{k}=0$. Together with ${W^{*}}^{⊤}W^{*}=I_{p}$ implied by Lemma A11, we can conclude that $W^{*}$ is a first-order stationary point of problem (1). Calculating the summation of the above inequalities from k=0 to N−1, we have

(A19) $$\begin{array}{cc} \; & \sum_{i=0}^{k}\frac{\bar{\eta }}{4}{∥D(W^{i})∥}_{F}^{2}\leq h\left(W^{0}\right)-h\left(W^{k}\right)<h\left(W^{0}\right)-\underset{\left|\right|W^{⊤}W-I_{p}{\left|\right|}_{F}^{2}\leq \delta }{\inf}h\left(W\right)\\ < & \underset{\left|\right|W^{⊤}W-I_{p}{\left|\right|}_{F}^{2}\leq \delta }{\sup}\tilde{h}\left(W\right)-\underset{\left|\right|W^{⊤}W-I_{p}{\left|\right|}_{F}^{2}\leq \delta }{\inf}\tilde{h}\left(W\right)+\frac{\beta }{4}\left(\left|\right|{W^{0}}^{⊤}W^{0}-I_{p}{\left|\right|}_{F}^{2}-\left|\right|{W^{k}}^{⊤}W^{k}-I_{p}{\left|\right|}_{F}^{2}\right)\\ \leq & 2m{∥Y∥}_{F}^{m}+\frac{\beta \delta }{2}, \end{array}$$

showing that $\underset{k\to +\infty }{lim inf}{∥D(W^{k})∥}_{F}=0$, which further implies that $D(W^{*})=0$. By Lemma A13, $D(W^{*})=0$ implies that $W^{*}$ is a first-order stationary point of (1). Moreover, by (A19), we have that

$$\min_{0\leq i\leq k}{∥D(W^{i})∥}_{F}^{2}\leq \frac{1}{k+1}\sum_{i=0}^{k}{∥D(W^{i})∥}_{F}^{2}\leq \frac{8m{∥Y∥}_{F}^{m}+2\beta \delta }{\bar{\eta }\left(k+1\right)},$$

and complete the proof. □

## Appendix C. Additional Experimental Results

In this section, we propose some additional numerical experiments. Figure A1 shows the top 12 basis computed from the testing instances in Section 4.1 by PenNMF. As described in [17], the top bases are those with the largest coefficients in terms of $\ell_{1}$-norm.
